# Characterization of Genomic Inheritance of Intergeneric Hybrids between *Ascocenda* and *Phalaenopsis* Cultivars by GISH, PCR-RFLP and RFLP

**DOI:** 10.1371/journal.pone.0153512

**Published:** 2016-04-07

**Authors:** Wen-Lin Liu, Huei-Chuan Shih, I-Szu Weng, Ya-Zhu Ko, Chi-Chu Tsai, Chang-Hung Chou, Yu-Chung Chiang

**Affiliations:** 1 Kaohsiung District Agricultural Research and Extension Station, Pingtung 900, Taiwan; 2 Department of Nursing, Meiho University, Pingtung 912, Taiwan; 3 Department of Biological Sciences, National Sun Yat-sen University, Kaohsiung 804, Taiwan; 4 National Pingtung University of Science and Technology, Pingtung 912, Taiwan; 5 Research Center for Biodiversity, China Medical University, Taichung 404, Taiwan; 6 Department of Biomedical Science and Environment Biology, Kaohsiung Medical University, Kaohsiung 807, Taiwan; National Cheng-Kung University, TAIWAN

## Abstract

**Background:**

The intergeneric hybrids between *Ascocenda* John De Biase ‘Blue’ and *Phalaenopsis* Chih Shang's Stripes have been generated to introduce the blue color into the *Phalaenopsis* germplasm in prior study. In order to confirm the inheritance in hybrid progenies, genomic *in situ* hybridization (GISH) and restriction fragment length polymorphism (RFLP) analysis were conducted to confirm the intergeneric hybridization status.

**Methods/Results:**

GISH analysis showed the presence of both maternal and paternal chromosomes in the cells of the putative hybrids indicating that the putative hybrid seedlings were intergeneric hybrids of the two parents. Furthermore, twenty-seven putative hybrids were randomly selected for DNA analysis, and the external transcribed spacer (ETS) regions of nrDNA were analyzed using polymerase chain reaction-restriction fragment length polymorphism (PCR-RFLP) and RFLP analyses to identify the putative hybrids. RFLP analysis showed that the examined seedlings were intergeneric hybrids of the two parents. However, PCR-RFLP analysis showed bias to maternal genotype.

**Conclusions:**

Both GISH and RFLP analyses are effective detection technology to identify the intergeneric hybridization status of putative hybrids. Furthermore, the use of PCR-RFLP analysis to identify the inheritance of putative hybrids should be carefully evaluated.

## Introduction

Comprising approximately 35,000 species, Orchidaceae is considered the largest family of flowering plants in the world [1]. Vandaceous orchids include *Vanda*, *Ascocenda*, *Phalaenopsis*, *Renanthera*, *Rhynchostylis* and *Aerides*, which are characterized by a monopodial growth habit and are mainly found in tropical Asia [2]. Although most intergeneric hybridizations in orchids fail due to irregular meiosis resulting from poor homology of the parental genomes [3,4], intergeneric hybridization among vandaceous plants is possible [5]. *Ascocenda* John De Biase ‘Blue’ is an intergeneric hybrid between *Ascocentrum* and *Vanda* species [6]. The cultivar is famous for its blue flowers and has been propagated and commercialized. *Phalaenopsis* is one of the most popular and ecologically important orchids worldwide [7,8] with huge interspecific hybrids [9]. *P*. Chih Shang's Stripes is an interspecific hybrid, and its genetic background includes species of *P*. *amboinensis*, *P*. *lueddemanniana*, *P*. *amabilis*, *P*. *aphrodite*, *P*. *equestris*, *P*. *schilleriana*, *P*. *sanderiana*, and *P*. *stuartiana* [6]. To date, more than 45,000 *Phalaenopsis* cultivars have been registered in the orchid database of Royal Horticultural Society (RHS) [10]. Intergeneric hybrids between *A*. John De Biase ‘Blue’ and *P*. Chih Shang's Stripes have been generated to introduce the blue color into the *Phalaenopsis* germplasm in prior study [11]. Hence, in order to confirm the inheritance in hybrid descendants, the various techniques including GISH, PCR-RFLP and RFLP are used to evaluate the genetic inheritance of these hybrids.

Apomixis is a unique type of asexual reproduction in many plant species that results in the formation of seeds that are genetically identical to the female parent, reflecting seed formation without fertilization. Apomixis has been found in a few agriculturally important crop species, such as citrus, apple, mango, and orchid [12]. Apomixis can easily be induced by interspecific and intergeneric hybridization [13–15] and detected by modern technology such as GISH and molecular markers [11]. Hence, this present study investigated the genetic inheritance of putative hybrids derived from the artificial hybridization of *A*. John De Biase ‘Blue’ (female parent) and *P*. Chih Shang's Stripes (male parent) based on GISH, PCR-RFLP and RFLP analyses of nrDNA.

Both genomic *in situ* hybridization (GISH) and restriction fragment length polymorphism (RFLP) analyses are powerful molecular analytical techniques. GISH was developed by Schwarzacher *et al*. [16] and has been used to detect foreign chromosomes and large DNA segments in interspecific or intergeneric hybrids as well as to analyze chromosome pairing activity, translocation breakpoints, and the genomic composition of polyploid plants [16–18]. RFLP analysis was developed by Botstein et al. [19] and has been widely used in the development of genetic markers and linkage maps [20]. RFLP has also been used to demonstrate the maternal inheritance of cpDNAs in both interspecific hybrids of *Phalaenopsis* [21]. In addition, a modified type of RFLP having the advantages of both RFLP and PCR, PCR-RFLP, has been used to develop high-fidelity, high-efficiency molecular markers that require a lower concentration of DNA [22]. In higher plants, nuclear ribosomal DNA (nrDNA) repeat units include the 18S, 5.8S, and 26S rRNA genes, which are separated by several spacers such as non-transcribed spacers (NTS), external transcribed spacers (ETS), and internal transcribed spacers (ITS) [23]. Both the internal and external transcribed spacers (ITS and ETS) have been widely used to infer phylogenetic relationships in plants [24–27]. Recently, PCR-RFLP analysis of nrDNA has also been used for cultivar identification in intergeneric and interspecific hybrids [11,28–33]. Therefore, in this study, we will apply the GISH, PCR-RFLP and RFLP identification techniques for investigating the intergeneric hybridization status between *A*. John De Biase ‘Blue and *P*. Chih Shang's Stripes.

## Results

### Genome analysis via GISH

**Fig 1:**
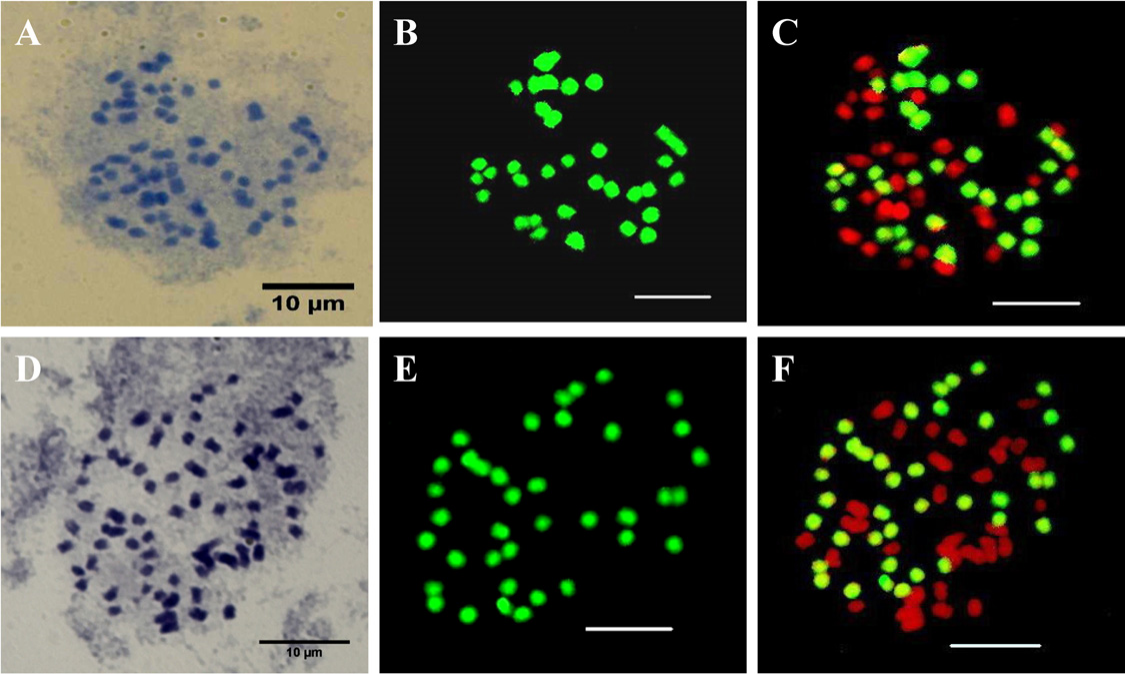
GISH of somatic metaphase chromosomes from intergeneric hybrids between *Ascocenda* John De Biase ‘Blue’ (♀) and *Phalaenopsis* Chih Shang's Stripes (♂). Giemsa-stained chromosomes before GISH (A, D) and after GISH using total DNA from *Phalaenopsis* Chih Shang's Stripes, as revealed with FITC (B, E). A composite image of chromosomes counterstained with propidium iodide (red) and showing FITC signals (green) (C, F). Scale bar: 10 μm.

Giemsa staining showed that *A*. John De Biase ‘Blue’ and *P*. Chih Shang's Stripes were triploid (2*n* = 3*x* = 57) and tetraploid (2*n* = 4*x* = 76), respectively (Fig 1A and 1D). The putative hybrids were derived from using *A*. John De Biase ‘Blue’ as the female parent and *P*. Chih Shang's Stripes as the male parent, and the number of somatic chromosomes in the hybrids varied from 3x + 5 to 3x + 10. Near-triploids were predominant. The accumulation of heterochromatin positively correlated with chromosome size [34–35]. The chromosomes size of *Phalaenopsis* species are highly variable with large (>2.5 μm), medium (2–2.5 μm) and small (<2.0 μm) chromosomes [34]. In this study, the chromosome size of the male parent (*P*. Chih Shang's Stripes) was either 2–2.5 μm or <2.0 μm. The chromosome size of the female parent (*A*. John De Biase ‘Blue’) varied between>2.5 μm, 2–2.5 μm, or <2.0 μm. By contrast, the male parent (*P*. Chih Shang's Stripes) did not have chromosomes>2.5um but female parent (*A*. John De Biase ‘Blue’) had chromosomes>2.5um. The result of giemsa staining reveals that the chromosome size of hybrids were 2–2.5 μm in which four chromosomes, and the others belongs to size of <2.0 μm (Fig 1).

Approximately 15 samples were examined by GISH analysis. GISH analysis showed that chromosomes derived from both the female and male parents could be found in the hybrid seedlings. One hybrid seedling contained 28 chromosomes derived from *A*. John De Biase ‘Blue’ (stained with PI) and 34 chromosomes derived from *P*. Chih Shang's Stripes (stained with FITC) (Fig 1B). Another seedling contained 31 chromosomes derived from *A*. John De Biase ‘Blue’ (stained with PI) and 36 chromosomes derived from *P*. Chih Shang's Stripes (stained with FITC) (Fig 1E). These results demonstrate that the assayed hybrid seedlings were intergeneric hybrids derived from the hybridization of *A*. John De Biase ‘Blue’ (female parent) and *P*. Chih Shang's Stripes (male parent). In addition, GISH analysis showed no chromosome recombination in the hybrids (Fig 1C and 1F).

### DNA analysis by PCR-RFLP

**Fig 2:**
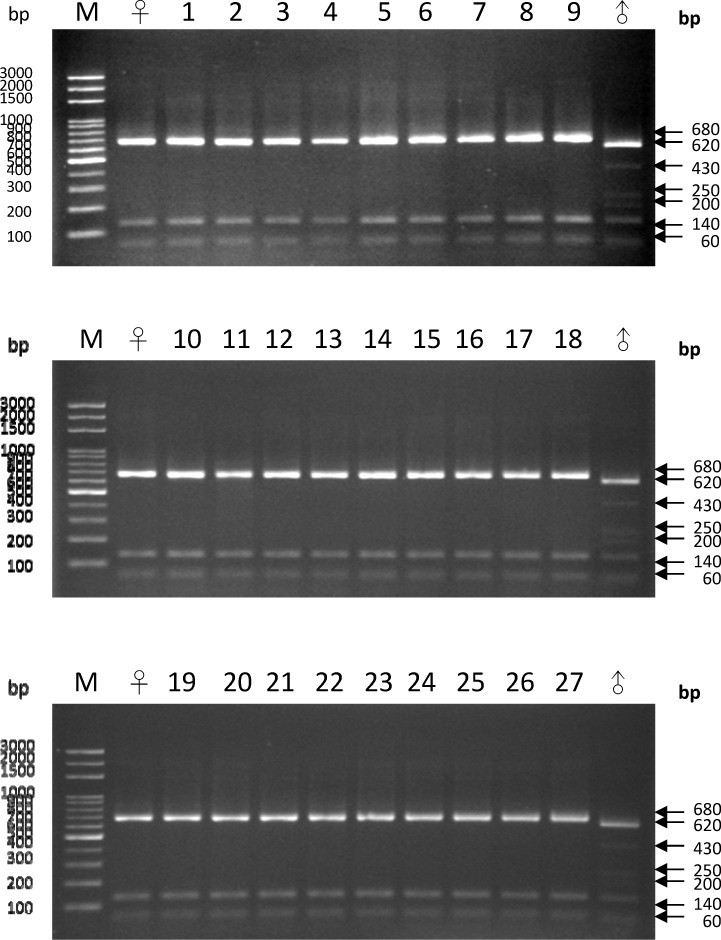
PCR-RFLP analysis of the external transcribed spacer (ETS) of nuclear ribosomal DNA. The PCR product of each sample was cut with the *Hae*III restriction enzyme. Lanes ♀ and ♂ represent the maternal parent (*Ascocenda* John De Biase ‘Blue’) and the paternal parent (*Phalaenopsis* Chih Shang's Stripes), respectively. Lanes 1–27 represent the 27 F1 hybrids. M: 100-bp DNA ladder marker.

All of the PCR amplification products from the rDNA ETS regions of *A*. John De Biase ‘Blue’, *P*. Chih Shang's Stripes and the 27 putative hybrid seedlings were approximately 880 bp. The PCR products were separately digested with *Hae*III and electrophoresed (Fig 2). After digestion, three DNA fragments of approximately 680, 140, and 60 bp were found in both *A*. John De Biase ‘Blue’ and in all 27 putative hybrids. By contrast, digestion of the *P*. Chih Shang's Stripes PCR product resulted in six DNA fragments of approximately 620, 430, 250, 200, 140, and 60 bp. Thus, the PCR-RFLP results do not reflect the biparental inheritance pattern of the ETS region in the putative hybrid seedlings but rather correspond to the DNA pattern of the female parent, *A*. John De Biase ‘Blue.’

### DNA analysis by RFLP

**Fig 3:**
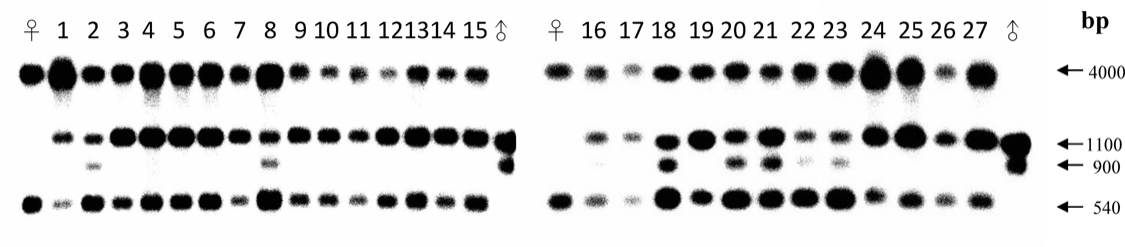
Southern blot analysis of the external transcribed spacer (ETS) patterns for each sample using total genomic DNA that was cut with the *Xba*I and *Dra*I restriction enzymes. Lanes ♀ and ♂ represent the maternal parent (*Ascocenda* John De Biase ‘Blue’) and the paternal parent (*Phalaenopsis* Chih Shang's Stripes), respectively. Lanes 1–27 represent the putative F1 hybrids.

RFLP analysis of the ETS region was conducted to further determine the inheritance of the ETS regions in the 27 putative hybrids. Total genomic DNA from *A*. John De Biase ‘Blue,’ *P*. Chih Shang's Stripes, and the 27 putative hybrids was separately digested with both *Xba*I and *Dra*I and hybridized with two different probes derived from the ETS regions of both *A*. John De Biase ‘Blue’ and *P*. Chih Shang's Stripes. After *Xba*I and *Dra*I digestion of the total DNA, two DNA fragments of approximately 4,000 and 540 bp were observed for *A*. John De Biase ‘Blue,’ whereas two bands of approximately 900 and 1,100 bp were observed for *P*. Chih Shang's Stripes. Two different RFLP DNA patterns were observed in the 27 putative hybrids. An RFLP pattern that included DNA fragments of 4,000, 1,100, and 540 bp was observed for 20 putative hybrids. The seven other putative hybrids (samples 2, 8, 18, 20, 21, 22, and 23) contained DNA fragments of 4,000, 1,100, 900, and 540 bp (Fig 3). These results are indicative of biparental inheritance of the ETS region. RFLP analysis showed that of the 27 putative hybrids, seven showed two bands representing the ETS region inherited from the male parent (the 1,100- and 900-bp fragments), and the other 20 hybrids showed a single type of ETS region (the 1,100-bp fragment) (Fig 3). These RFLP results demonstrate the biparental inheritance pattern of the ETS region in the putative hybrid seedlings.

### Sequencing and inverse PCR

**Fig 4:**
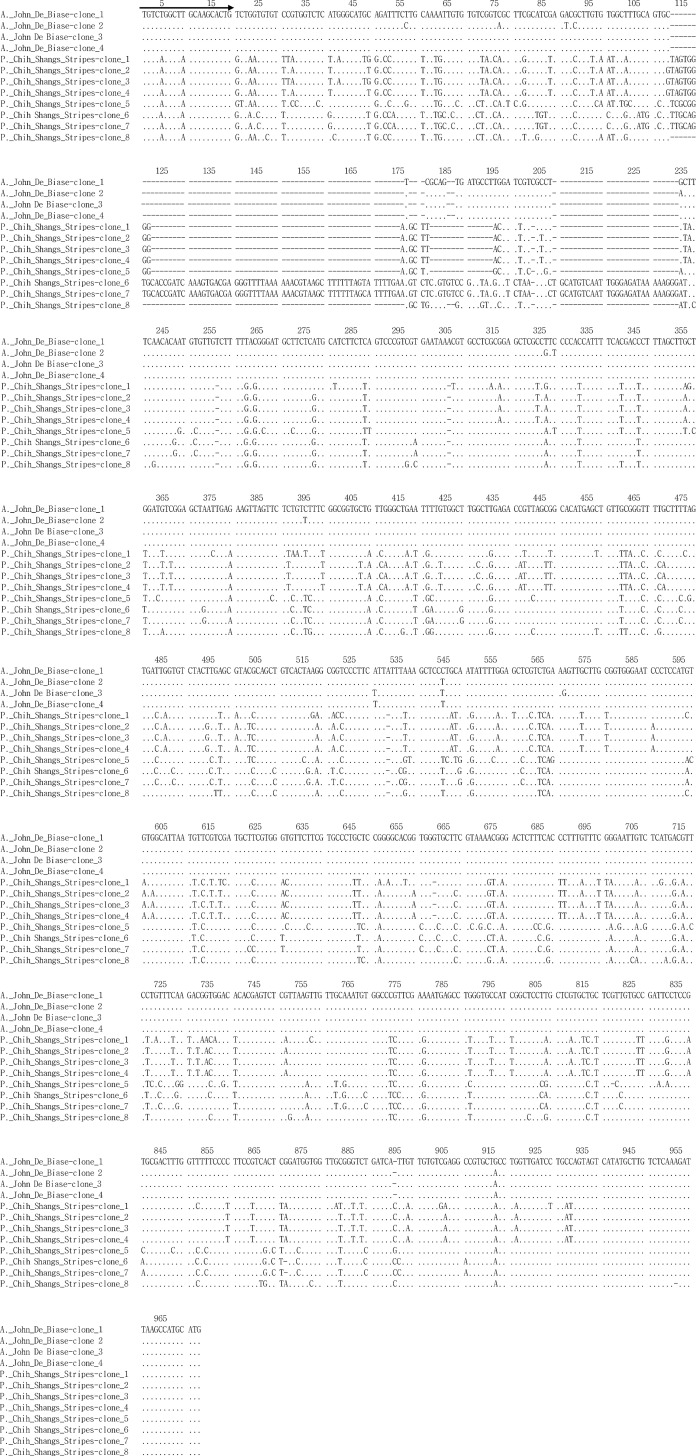
The alignment of validated ETS sequence derived from both the male parent (*Phalaenopsis* Chih Shang's Stripes) and female parent (*Ascocenda* John De Biase ‘Blue’). The Arrow represents the location of the forward primer for the ETS amplification by PCR.

The ETS PCR products were amplified from both the female and male parents. Seventeen clones from each sample were randomly selected for sequencing. In the male parent, *P*. Chih Shang's Stripes, eight types of ETS sequences were found (Fig 4), and RFLP analysis with *Xba*I and *Dra*I digestion showed that they could be classified into two types of patterns (Fig 3). In the female *A*. John De Biase ‘Blue,’ there were four types of ETS sequences, and RFLP analysis with *Xba*I and *Dra*I digestion showed that they could only be classified into one type of pattern (Fig 3). In addition, iPCR validation showed two mismatches at the 5’ forward primer binding site in the ETS region of the male parent, *P*. Chih Shang's Stripes. All of the validated ETS sequences derived from both the male and female parents were aligned and are shown in Fig 4.

## Discussion

Generally, the number of chromosomes in diploid *Phalaenopsis* and *Ascocenda* species is 2n = 2x = 38 [3,36–37], although the sizes of *Phalaenopsis* chromosomes vary greatly [34]. Based on its genealogy, the genetic background of the male parent *P*. Chih Shang's Stripes included *P*. *amboinensis*, *P*. *lueddemanniana*, *P*. *amabilis*, *P*. *aphrodite*, *P*. *equestris*, *P*. *schilleriana*, *P*. *sanderiana*, and *P*. *stuartiana* [6]. Kao et al. [34] examined the karyotypes of several *Phalaenopsis* species and demonstrated that the sizes of their chromosomes are highly variable and can be classified into three types: < 2.0 μm, 2–2.5 μm, and > 2.5 μm. Of the *Phalaenopsis* chromosomes in the putative hybrids, four are medium-sized (2–2.5 μm) and may have been inherited from *P*. *amboinensis* and *P*. *lueddemanniana*. The remaining *Phalaenopsis* chromosomes are small (< 2.0 μm) and may have been inherited from *P*. *amabilis*, *P*. *aphrodite*, *P*. *equestris*, *P*. *schilleriana*, *P*. *sanderiana*, or *P*. *stuartiana* [34,36]. The accumulation of heterochromatin has been shown to be positively correlated with an increase in chromosome size [34–35]. General concept, triploid plants are generally not useful for breeding because of their frequent sterility. However, there are triploids that have been used successfully as parents for breeding [38]. This study demonstrates that the female parent, *A*. John De Biase ‘Blue,’ is triploid and can be used as the female parent for hybridization with *P*. Chih Shang's Stripes to obtain F1 progeny via embryo rescue [11]. The F1 hybrids were predominantly near-triploid, as shown by chromosome inspection. This result is different from that of the cross between diploid and tetraploid taxa of *Lilium*, which generated progeny that were predominantly pentaploid or near-pentaploid [39]. In addition, when the parental chromosome number is imbalanced (i.e., the parent is triploid or pentaploid), irregularities may easily occur during meiosis, leading to the imbalanced distribution of hybrid individuals [40]. This phenomenon can explain the progeny with irregular numbers of chromosomes observed in this study, i.e., they resulted from an imbalanced parent.

GISH has proven to be a useful technique to distinguish genetically recombined genomes and assess the genomic relationships between different hybrid plants [16,18,41]. In GISH analysis, the addition of a large amount of blocking DNA can increase the probe specificity and is usually required for hybrids derived from two parents that have a close relationship [42], such as has been described in *Triticeae* species [42] and *Phalaenopsis* species [43]. In this study, chromosomes derived from the female parent, *A*. John De Biase ‘Blue’, or from the male parent, *P*. Chih Shang's Stripes, can be clearly distinguished without the use of blocking DNA. These results indicate that the parents do not have a close genetic relationship, which is in agreement with their classification into two separate genera [1]. GISH analysis showed that chromosomes derived from both the female and male parents could be found in the hybrid seedlings. In addition, GISH analysis showed no obvious homologous chromosome recombination in the intergeneric hybrids, likely resulting from the low sequence similarity of the homologous chromosomes of both parents [44–46]. Only a few GISH studies have documented chromosome recombination in intergeneric hybrids, including *Festuca pratensis* × *Lolium multiflorum* [47] and *Lycopersicon esculentum* × *Solanum tuberosum* [48]. Compared with intergeneric hybrids, in interspecific hybrids, homologous chromosome recombination usually occurs at a higher frequency [39,49].

Both cytoplasmic and nuclear inheritance are complex phenomena that can be divided into three modes: maternal, paternal and biparental. Usually, nuclear inheritance is biparental, and the cytoplasm is generally inherited uniparentally [50]. In previous studies, nrDNA has proven to be a useful molecular marker [51–54]. nrDNA can be easily amplified using universal primers and exhibits relatively high levels of phylogenetically informative sequence variation that can differentiate between closely related taxa, such as in the ITS regions [32]. In this study, we used the ETS regions of nrDNA in both PCR-RFLP and RFLP analyses to identify the putative hybrids as intergeneric hybrids and to evaluate their mode of inheritance. If concerted evolution occurs, the same repeat sequences will become homogenized by mechanisms such as high-frequency unequal crossing over or gene conversion [55–56], as has been described for *Gossypium* [51], *Cardamine* [57], *Rheum* [54] and *Acer* [58]. In this study, biparental inheritance of nrDNA in the intergeneric hybrids was shown, representing the occurrence of non-concerted evolution. This phenomenon was also found in several studies of biparental nrDNA inheritance in first-generation hybrids, such as in *Paeonia* [59], *Musa* [28], *Nicotiana tabacum* [60–62], and *Hordeum vulgare* L. × *Hordeum spontaneum* L. [63]. However, the maternal inheritance of the ETS regions demonstrated by the PCR-RFLP analysis is very unusual (Fig 2) and indicates that the seedlings were derived from apomixis. This result is not consistent with the PCR-RFLP analysis of the nrDNA ITS region in a previous study [11]. Because both the ETS and ITS regions are located in the same nrDNA repeat unit [55–56], they should have same mode of inheritance. Consequently, RFLP analysis of the ETS regions was further analyzed. RFLP analysis indicated that the ETS region of the hybrids was biparentally inherited. Therefore, the maternal inheritance pattern of the ETS region in the F1 hybrids might indicate biased PCR amplification in the ITS region in the PCR-RFLP analysis, as described by Diaz and Sabino [64]. Generally, biased PCR amplification is caused by disparate G+C contents [65], the content of the template DNA [66], or the match with the primer binding site [65]. The male and female parents are tetraploid and triploid, respectively. Thus, distinct template DNA contents can be ruled out. In addition, the PCR-amplified ETS regions of both parents were separately sequenced by T-vector-based cloning (Fig 4). The sequencing results indicated that there are eight different ETS sequence clones in the male parent that represent seven different G+C contents (43.0, 43.1, 43.2, 43.4, 52.4, 53.1, 53.2, and 57.0%). By contrast, there are four different ETS sequence clones in the female parent that represent four different G+C contents (51.5, 51.8, 51.9, and 52.0%). Although the G+C contents of several ETS sequences in the male parent are lower than those of the female parent, PCR-RFLP cannot clearly detect the ETS sequences of the male parent in the hybrids. Therefore, a G+C content leading to biased PCR amplification can be ruled out. In addition, the ETS sequences indicated two mismatches at the 5’ forward primer binding site of the ETS region in the male parent (Fig 4), likely leading to bias towards the female ETS region during PCR amplification.

Furthermore, two types of ETS regions inherited from the male parent were identified in the F1 intergeneric hybrids; samples 2, 8, 18, 20, 21, 22, and 23 inherited two DNA patterns that were derived from the male parent (Fig 3). This result can be explained by the tetraploidy of the male parent. General genetic concepts predict that the pollen will be diploid, leading to the hybrids having one (homogenous) or two (heterogeneous) types of male parent-derived ETS regions.

## Conclusions

To sum up, GISH and RFLP analyses are useful for identifying intergeneric hybrids derived from *Ascocenda* John De Biase ‘Blue’ (female parent) and *Phalaenopsis* Chih Shang’s Stripes (male parent) and evaluating the genetic inheritance of these hybrids. The results showed that the GISH and RFLP method can effectively identify all F1 hybrid seedlings are intergeneric hybrids. Moreover, the use of PCR-RFLP analysis to assess the inheritance of the putative hybrids should be carefully evaluated.

## Materials and Methods

### Plant materials

The plant materials used in this study consisted of F1 intergeneric hybrid seedlings derived from an *Ascocenda* John De Biase ‘Blue’ female parent and a *P*. Chih Shang's Stripes male parent. The intergeneric hybrids were obtained via embryo rescue [11]. All plants were cultivated in the greenhouse of the Kaohsiung District Agricultural Research and Extension Station, COA, Pingtung, Taiwan.

### Chromosome preparation

Young root tips were collected from the two parents and the putative hybrids. They were then treated with 2 mM 8-hydroxyquinoline on a shaker (100 cycles min-1) at 18°C for 5 hr, fixed in Carnoy’s solution (3:1, v/v, ethanol:glacial acetic acid) and stored at 4°C for 24 hr. The root tips were then hydrolyzed in 1 N HCl at 60°C for 5–10 min, treated with 1% pectinase for 1 hr, crushed in 45% acetic acid on microscope slides that had been pretreated with Vectabond (Vector Laboratories, UK), and covered with cover glasses. The slides were frozen in liquid nitrogen, and the cover glasses were removed with a razor blade. The slides were then air-dried, and stained with Giemsa stain for 5 min. The slides were destained with formamide and stored in a dessicator at -20°C until use. Non-overlapping chromosomes were selected for the GISH experiments.

### Genomic *in situ* hybridization

More than 15 samples were examined by GISH analysis. Genomic DNA was extracted from young leaves of the two parents and the putative hybrids using the CTAB method [67]. DNA was then labeled with DIG-11-dUTP (Roche Ltd.) via nick translation and post-fixed in 4% paraformaldehyde for 10 min. The chromosomal DNA was denatured in 70% formamide in 2× SSC at 70°C for 2.5 min and dehydrated through an ethanol series at 4°C. The hybridization mixture consisted of 50% formamide, 10% dextran sulfate, 2× SSC, 0.1% SDS, and 50 ng/μl of DNA probe. Hybridization was performed at 37°C overnight. The slides were washed in 20% formamide in 0.1× SSC at 42°C for 10 min, in 2× SSC at 42°C for 10 min, and in 2× SSC at room temperature for 5 min. The labeled probe was detected with fluorescein-conjugated antibodies (Roche Molecular Biochemicals), and the chromosomes were counterstained with propidium iodide (PI). The prepared materials were observed by confocal fluorescence microscopy (Leica CTR 6500). Furthermore, the GISH analysis in this study did not use the blocking DNA, since chromosomes derived from the female parent, *A*. John De Biase ‘Blue’, or from the male parent, *P*. Chih Shang's Stripes, can be clearly distinguished without the use of blocking DNA.

### PCR-RFLP analysis

**Fig 5:**
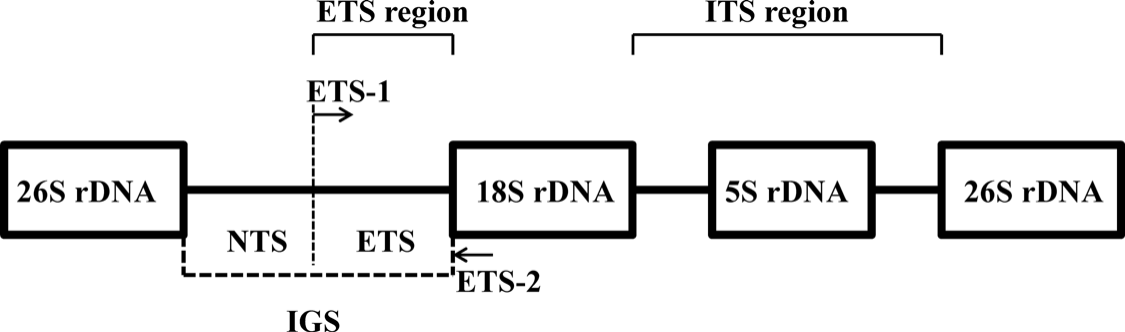
The gene structure of the nuclear ribosomal DNA. The arrows indicate the positions of the primers used in this study.

External transcribed spacer (ETS)-specific primers for PCR amplification were designed based on reference sequences from the GenBank database, including maize (GenBank accession no. X03990), wheat (X07841), rice (X54194), *Setaria italica* (AB197128), *Linum* (EU307117), and *Sarcochilus falcatus* (AF321598). The primers were ETS-1 (5’-TGTCTGGCATGCAAGCACTG-3’) and ETS-2 (5’-CATGCATGGCTTAATCTTTGAGAC-3’) (Fig 5). Each PCR amplification was performed in a 50-μL volume containing 1 μL of template DNA, 25 μL of SuperTherm Gold DNA Polymerase Mix (2X PCR Master Mix), and 0.5 pmoles of each primer. The PCR conditions were as follows: 94°C for 5 min; 35 cycles of 94°C for 25 sec, 60°C for 30 sec, and 72°C for 30 sec; and 72°C for 7 min. The amplified PCR products were digested with *Hae*III at 37°C for 1 hr and separated by electrophoresis at 50–80 V in 1.5% agarose gels with 0.5 μg/mL ethidium bromide. The patterns of the digested PCR products were visualized under UV light.

### RFLP analysis

Genomic DNA was isolated from fresh leaves using the CTAB method [67]. One microgram (1 μg) of genomic DNA was digested using *Xba*I and *Dra*I, separated on a 1.0% agarose gel by electrophoresis, and transferred to a Hybond N+ nylon membrane (Amersham Biosciences, Buckinghamshire, UK) via the capillary method. Probe preparation, hybridization, and detection were conducted using a DIG High Prime DNA Labeling and Detection Starter Kit II (Roche Applied Science, IN, USA) according to the manufacturer’s instructions. The probe was prepared via PCR amplification of the ETS region using primers ETS-1 and ETS-2 (Fig 5). The PCR thermal cycling conditions were as follows: at 94°C for 5 min; 35 cycles of 94°C for 25 sec, 64°C for 25 sec, and 72°C for 30 sec; and 72°C for 7 min. The chemiluminescent signal was captured on X-ray film (Kodak 4000R).

### Sequencing and inverse PCR

The PCR products were recovered using a MinElute PCR Purification kit with the provided spin columns (Qiagen, Germany). The recovered PCR products were ligated into a T-vector (Promega Co., USA), and the resulting recombinants were transformed into *Escherichia coli*. The plasmid DNA was purified with a Qiagen Spin Miniprep kit (Qiagen, Germany) and sequenced with vector-specific primers (SP6 and T7) according to the manufacturer’s recommendations. To validate the sequences of the ETS primer binding sites in all of the studied samples, inverse PCR (iPCR) was conducted as described by Ochman et al. [68]. Sequence alignment was performed using the ClustalW multiple alignment tool in BioEdit [69].
